# Deep wound infection after a trochanteric fracture internal fixation presenting with hip dislocation: a case report

**DOI:** 10.1186/1757-1626-3-19

**Published:** 2010-01-12

**Authors:** Matheus Tzurbakis, Emmanouil Morakis, Georgios Mouzopoulos, Nikolaos Lasanianos, Ioannis Georgilas

**Affiliations:** 1 1st Orthopaedic Department of 'Evaggelismos' General Hospital, Athens, Greece

## Abstract

We report a rare case of posterior hip dislocation after a low energy trauma. The patient sustained a trochanteric fracture in the same hip six months ago, which was fixed using a sliding hip screw and had healed. At surgery a deep wound infection was found and a methicillin-resistant Staphylococcus epidermidis (MRSE) was cultured. After thorough debridement, an excisional arthroplasty was decided. The patient received specific intravenous antibiotics and after six weeks a total hip arthroplasty was done. In three years follow-up the patients presented with a fully functional hip without any signs of infection. Hip dislocation after a trochanteric fracture internal fixation is rare complication associated with high morbidity and mortality. Infection eradication and a second stage arthroplasty can be life and limb saving.

## Introduction

In our aging western societies hip fracture incidence increases. Trochnanteric fractures account about half of all hip fractures in elderly people. Maybe the most widely used surgical method for trochanteric fracture stabilization is the use of a sliding hip screw. It is a surgical method with good results and few complications. Internal fixation complications include varus collapse with cutout of the compression screw, disassembly of the lag screw from the side plate, fracture nonunion, osteonecrosis of the femoral head and wound infection. Hip dislocation following a trochanteric hip fracture treated with dynamic hip screw internal fixation is an extremely rare complication. There are few reports of this complication after valgus fixation of the fracture[1], haemarthrosis of the hip joint [2] or hip septic arthritis [3]. We present a case of hip dislocation in a patient treated for a trochanteric fracture with a sliding hip screw internal fixation, complicated with deep wound infection.

## Case report

**Figure 1:**
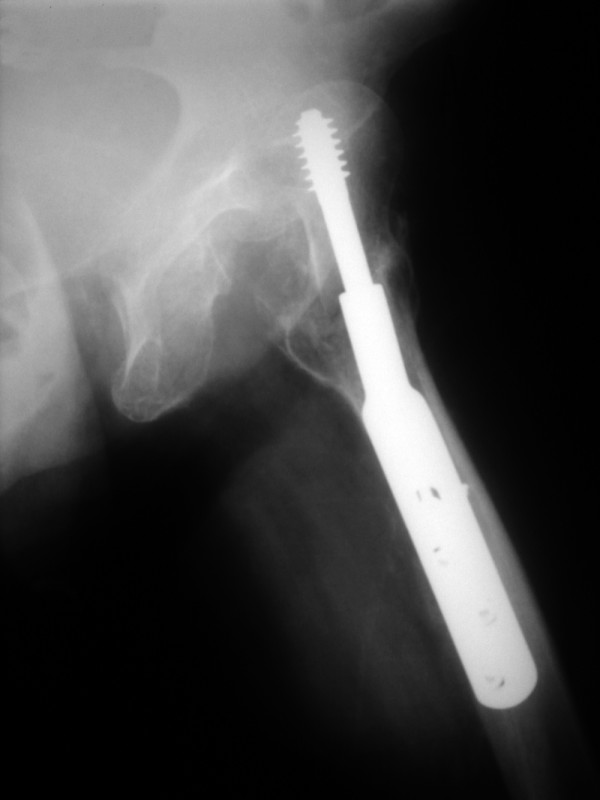
**X-ray of the left hip demonstrating a posterior hip dislocation**.

**Figure 2:**
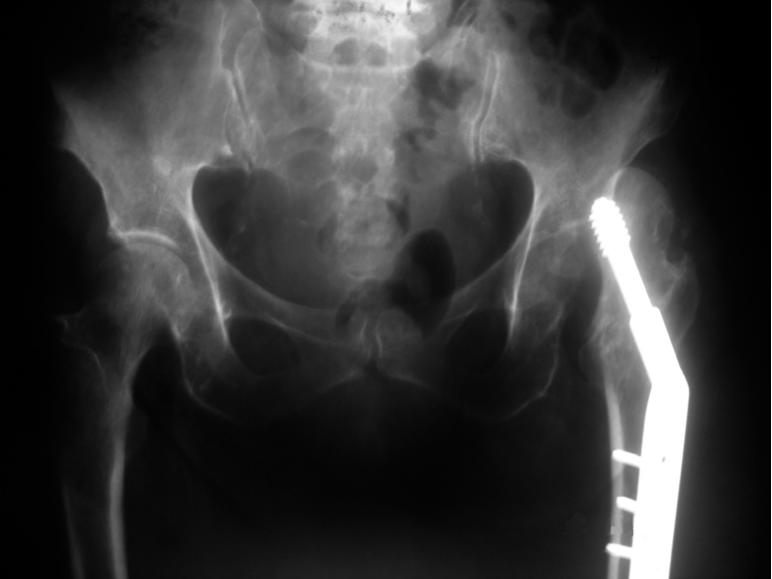
**X-ray demonstrating a posterior hip dislocation while the old trochanteric fracture has healed**.

**Figure 3:**
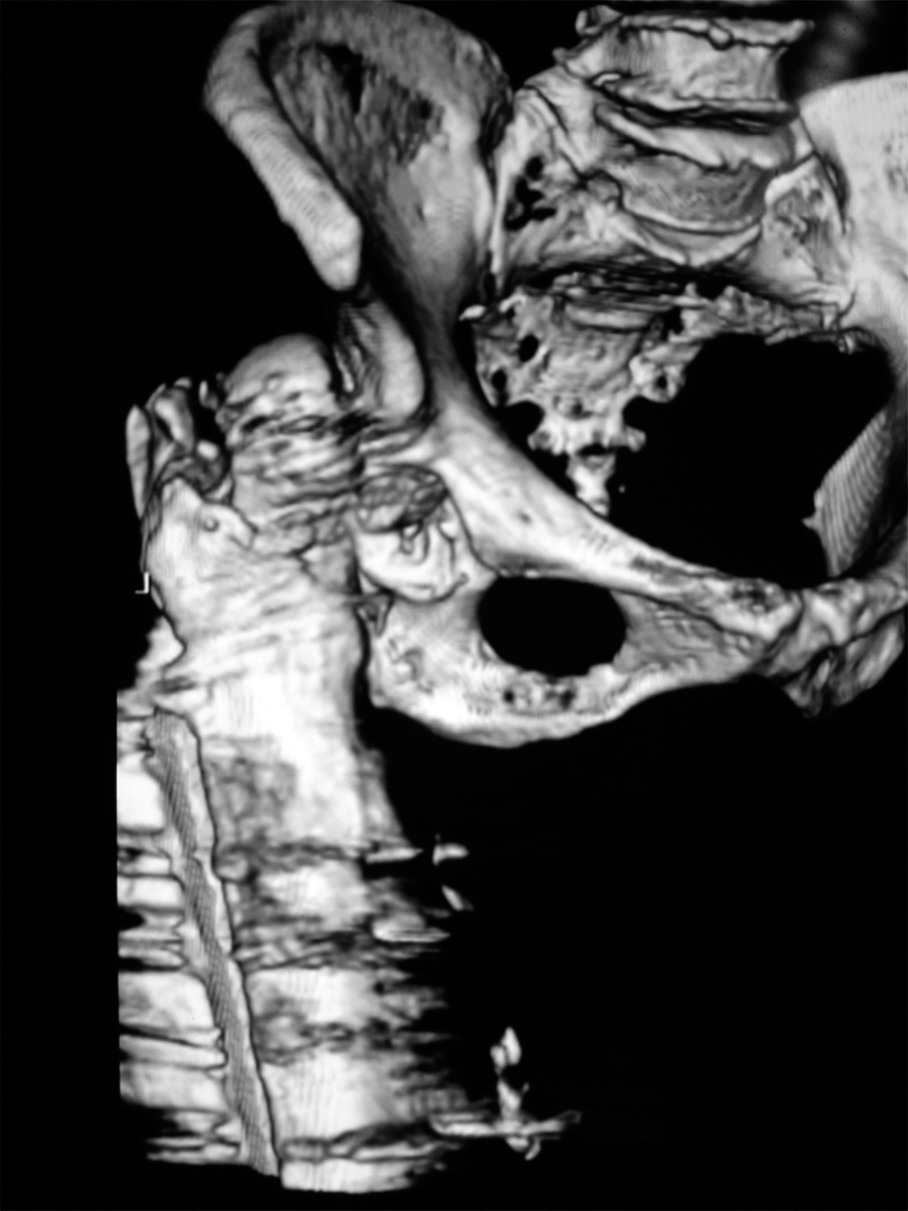
**A 3-dimentional reconstructed computer tomography demonstrating the hip dislocation**.

**Figure 4:**
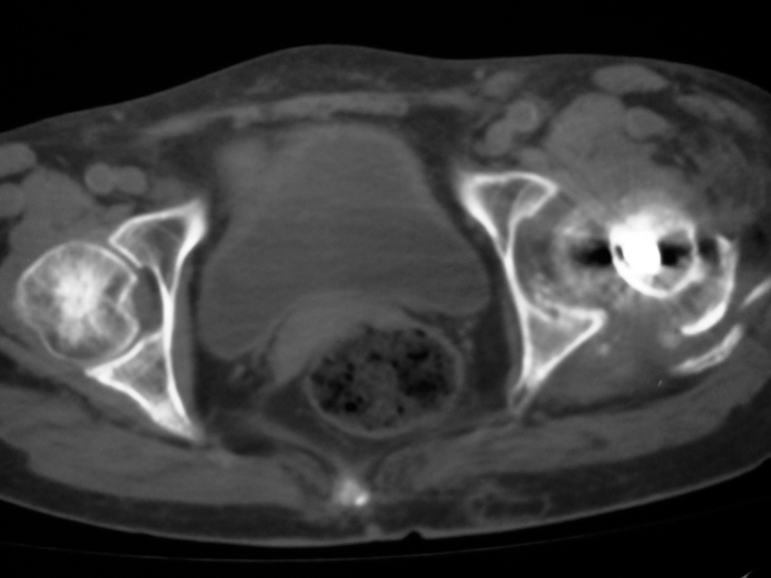
**Computer Tomography demonstrating the femoral head and posterior acetabular wall destruction**.

A 78-year-old female presented in the emergency department limping following a low-energy fall 24 hours ago. Her left limb was painful, shortened and internally rotated without any vascular or neurological compromise. She presented without fever, wound drainage, abscess formation or cellulitis. X-ray imaging performed revealed a left hip posterior dislocation (Fig 1 &2). Computer tomography imaging confirmed the diagnosis and didn't reveal an abscess formation (Fig. 3 &4). The patient sustained a left trochanteric fracture six months ago which was fixed using a sliding hip screw. Her postoperative period was without any complications and the fracture has united.

Her medical history revealed arterial hypertension, type 2 diabetes mellitus managed with insulin and hyperlipidemia. Laboratory tests revealed increased erythrocyte sedimentation rate (ESR = 114), increased C-reactive protein (CRP = 9.38) and increased leucocyte count (WBC = 14,830). Her physical examination and the rest of the laboratory tests (chest x-ray, urine analysis, blood & urine cultures, heart & abdominal ultrasound examination) didn't reveal another site of inflammation.

After the preoperative work-up the patient was transferred to the operating room to reduce the dislocated hip. A deep wound infection with tissue inflammation and necrosis was found with the left hip dislocated posteriorly. The internal fixation was stable and the trochanteric fracture had healed. Soft tissue debridement removing all infected and nonviable tissue was done. Fluid found and specimens of infected deep soft tissues were sent for immediate Gram's stain, culture and antibiotic sensitivity tests. The Gram's stain revealed numerous polymorphonuclear cells.

Since the fracture had healed, the hip joint was dislocated and inflamed with part of the femoral head eroded, an excisional arthroplasty was decided. The internal fixation implants were removed, the femoral head was osteotomized, and the medullary canal and the acetabulum were reamed. The femoral head and reaming products were sent for histological examination, which was positive for inflammation. The wound was thoroughly irrigated and closed over a suction drainage system.

A skeletal traction system from the tibial tuberosity was used to maintain muscle tension. Based on the results of the Gram's stain IV vancomycin and ciprofloxacin was administered. The wound specimens' cultures recovered a methicillin-resistant Staphylococcus epidermidis (MRSE) sensitive to vancomycin.

**Figure 5:**
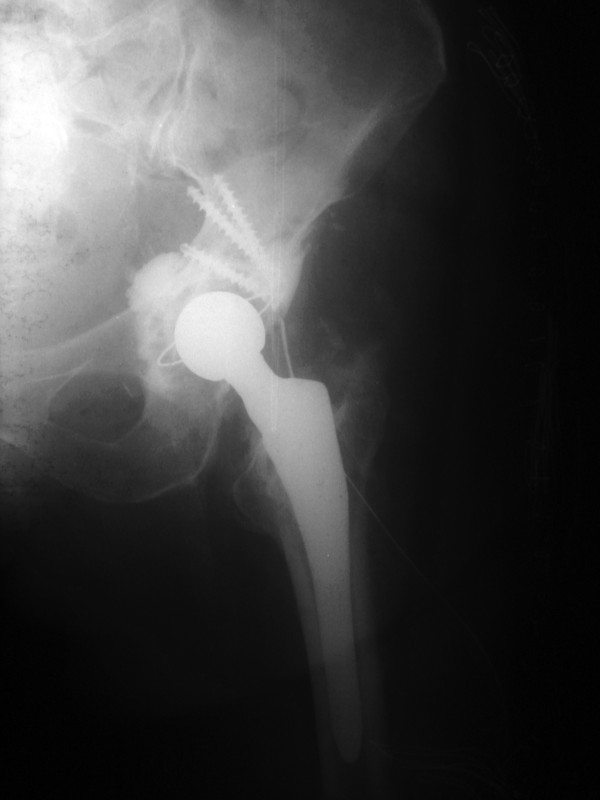
**Postoperative x-ray after the total hip arthroplasty**. A posterior-superior acetabular lip bone loss restored with hip autograft and antibiotic-impregnated cement fixed with three screws.

The patient was administered IV antibiotics for six weeks and the infection indices values returned to normal. Then a new operation was carried out. The tissues were redebrided and specimens sent for Gram's stain & culture. The wound showed no signs of infection and a total hip arthroplasty was performed. A cementless femoral component was implanted with an antibiotic-impregnated cemented acetabular component. Hip autograft with antibiotic-impregnated cement was fixed with three screws to restore a posterior-superior acetabular lip bone loss (Fig. 5).

The patient had a postoperative period without complications. In the follow-up, three years after the arthroplasty, she can walk without pain or limp, the hip wound shows no signs of infection and the laboratory results are within normal values.

## Discussion

Hip dislocation is usually associated in infants and children with cerebral palsy, developmental hip dysplasia and trauma. Even low-energy trauma can cause hip dislocation in infants and younger children because their periarticular structures are more flexible.

On the contrary, in adults hip dislocation is the result of direct force trauma to the thigh, usually high-energy trauma after a motor-vehicle accident or a fall from a significant height. Hip dislocation following a low-energy trauma is rare. There have been reported few cases of hip dislocation or subluxation in an adult following low-energy trauma complicated by myositis ossificans [4], an osteochondroma [5] or during a dance [6].

Hip dislocation following a trochanteric hip fracture treated with dynamic hip screw internal fixation is an extremely rare complication. This complication has been reported after valgus fixation of the fracture [1], sometimes with haemarthrosis of hip joint [7], hip capsule trauma [8] or hip septic arthritis[9].

Septic dislocation after internal fixation of trochanteric fractures has been reported for the first time by J.S. Speed & R.A. Knight[10]. Later, P.E.L. Evans reported three cases of septic dislocation after internal fixation of a trochanteric fracture[11]. In all of these cases the outcome was poor or fatal. Two patients died after four and six months respectively and during this time could not walk. The third patient was unable to walk again.

Deep wound infections after a trochanteric fracture complicated with hip dislocation are extremely rare but serious complications with poor outcome usually. In our case the patient had a low grade infection after the surgery for the trochanteric fracture since methicillin-resistant Staphylococcus (MRSA & MRSE) strains are usually hospital-acquired. MRSE is the most common pathogen isolated from infected prosthetic devices in orthopedic surgery which can colonize in a protective biofilm around the implant, especially in immunocompromised patients.

Our patient didn't complain for any evident clinical signs or symptoms of infection. This can be attributed to a low response of the immune system due to malnutrition and diabetes. Even if she had any hip pain or decreased range of motion, she probably attributed these symptoms to her fractured hip.

This low grade infection, in conjunction with her bad overall health status resulted in a hip soft tissue damage and hip joint laxity. So even after this low-energy fall she sustained a posterior hip dislocation through the infected and lax hip capsule.

In surgery the implants were removed and any soft tissue that seemed infected or necrotic was excised, in order to minimize bacterial burden. The femoral head was removed and the acetabulum reamed to ascertain that all possible bacterial contaminated sites were removed and to prepare for a secondary arthroplasty.

Total hip arthroplasty was done after all infection indices return to normal values. The result was a stable, completely functional and pain free hip joint. The patient returned fully functional to her previous life activities.

Life and limp salvage are first priorities in such cases. Thorough surgical debridement and specific antibiotic treatment is the mainstay of infection eradication. After complete remission of the infection, if the patient's health status permits it, a second stage arthroplasty is the best option to improve the patient's functional status.

Hip dislocation complicating a trochanteric hip fracture treated with a sliding hip screw is a rare complication. Nevertheless it is associated with increased morbidity and mortality, especially cases with hip infection. The surgeon must have a high index of suspicion when a patient with a history of hip surgery presents with hip complaints. The laboratory and imaging tests will help him in the differential diagnosis.

## Consent

Written informed consent was obtained from the patient for publication of this case report and accompanying images. A copy of the written consent is available for review by the Editor-in-Chief of this journal.

## Competing interests

The authors declare that they have no competing interests.

## Authors' contributions

All authors contributed to the patient's treatment and to the writing of the manuscript. All authors read and approved the final manuscript."
